# Paclitaxel Chemotherapy Disrupts Circadian Gene Transcription and Function of the Suprachiasmatic Nuclei in Female Mice

**DOI:** 10.1523/ENEURO.0061-25.2025

**Published:** 2025-09-18

**Authors:** Zoe M. Tapp, Melina M. Seng, Amiya K. Ghosh, Karl H. Obrietan, Leah M. Pyter

**Affiliations:** ^1^Institute for Behavioral Medicine Research, The Ohio State University, Columbus, Ohio 43210; ^2^Departments of Neuroscience, Wexner Medical Center, The Ohio State University, Columbus, Ohio 43210; ^3^Psychiatry and Behavioral Health, Wexner Medical Center, The Ohio State University, Columbus, Ohio 43210

**Keywords:** entrainment, fatigue, jet lag, molecular clock, phase response curve, SCN

## Abstract

Cancer patients experience circadian rhythm disruptions during and after chemotherapy that can contribute to debilitating side effects. It is unknown how chemotherapy mediates circadian disruptions and specifically the extent to which these disruptions occur at the level of the principal clock, the suprachiasmatic nuclei (SCN) of the hypothalamus. In the present study, we assessed how the commonly used chemotherapeutic, paclitaxel, impacts the SCN molecular clock and SCN-dependent behavioral adaptations to circadian challenges in female mice. Following a repeated chemotherapy regimen, we measured rhythmic SCN expression of molecular clock and circadian-associated transcripts. Paclitaxel chemotherapy disrupted the SCN molecular clock through abolished rhythmicity (*Bmal1*, *Nr1d2*) and damped rhythmic transcription (*Ciart*, *Dbp*, *Nr1d1*, *Per2*) of key molecular clock genes. We further determined chemotherapy-induced changes to SCN function by measuring circadian wheel running adaptations to a jet lag phase-delay or phase-advance paradigm and by generating a phase response curve (PRC). Chemotherapy did not alter re-entrainment to a 6 h phase-advance, but after a 6 h phase-delay, chemotherapy-treated mice had a more stable and robust circadian rhythm than vehicle-treated mice, possibly indicative of a weakened or decoupled SCN. In the PRC, chemotherapy blunted light-induced phase-shift delays during subjective night compared with vehicle controls, also indicative of disrupted SCN-dependent entrainment. Together, this work demonstrates that paclitaxel chemotherapy disrupts both the molecular clock and functional re-entrainment of the SCN that could cause or contribute to observed circadian rhythm disruptions after treatment. This research could help guide application of circadian-mediated therapies to mitigate side effects of chemotherapy.

## Significance Statement

Circadian rhythm disruption is a prevalent side effect of chemotherapy treatment, with up to half of patients with cancer exhibiting impaired circadian rhythms associated with treatment-induced side effects and decreased quality of life. Mechanisms contributing to chemotherapy-induced circadian disruption remain elusive, such as the extent to which the central clock, the suprachiasmatic nuclei (SCN) of the hypothalamus, is involved. Here, we assess the impact of the commonly used paclitaxel chemotherapy on the molecular clock of the SCN and SCN function. We find that paclitaxel chemotherapy robustly disrupts the molecular clock of the SCN associated with altered SCN-dependent behavioral adaptations. Understanding the extent to which chemotherapy alters SCN function will inform potential targets to reduce debilitating side effects of chemotherapy.

## Introduction

Chemotherapeutic cancer treatments lack specificity, resulting in debilitating off-target side effects both during and after treatment. One such side effect repeatedly reported following chemotherapy in both preclinical and clinical studies is circadian rhythm disruption (Amidi and Wu, 2022; Tapp et al., 2025). Nearly every living organism displays circadian rhythmicity to anticipate and adapt to their environment and ensure physiological efficiency (Francis et al., 2019; Perry et al., 2024). Indeed, many physiological processes that contribute to homeostasis, such as metabolism and sleep, are regulated in a circadian fashion (Lok et al., 2024). As such, disruption of circadian rhythms can have a range of deleterious health effects, from fatigue to disease pathogenesis (Sletten et al., 2020).

Circadian rhythms in mammals are dictated by the primary pacemaker, the suprachiasmatic nuclei (SCN) of the hypothalamus (Starnes and Jones, 2023). This small bundle of neurons contains autonomous pacemaker capabilities that maintain rhythmicity through a tightly regulated molecular clock attuned to environmental cues known as zeitgebers, the most salient of which is light. The molecular clock is comprised of intersecting transcriptional–translational feedback loops that together maintain an ∼24 h (i.e., circadian) rhythm at the cellular level and influences SCN neuronal activity and responsiveness to zeitgebers in a time-dependent manner (Ko and Takahashi, 2006). While the SCN maintains a rigid endogenous circadian rhythm, it is also flexible to adapt to changes in the environment, such as light information (Ashton et al., 2022). Importantly, nearly every cell in the body has a molecular clock, but the molecular clock within the SCN is unique in that it coordinates the rhythms of the rest of the body through both neuronal and humoral communication (Buijs et al., 2006).

Chemotherapy-induced circadian disruption is well documented in clinical populations, with up to half of patients with cancer presenting evidence of disrupted rhythms including arrhythmicity of key circulating hormones (i.e., glucocorticoids), disrupted daily activity rhythms, and sleep disturbances during or after chemotherapy treatment (Milanti et al., 2021; Amidi and Wu, 2022). The mechanisms driving these chemotherapy-induced circadian disruptions are poorly understood. Mouse models of chemotherapy treatment provide the opportunity to identify these mechanisms; however, to date, most experimental evidence of circadian disruption has been characterized in peripheral oscillators such as the liver, heart, and kidneys (Tapp et al., 2025) which are more accessible to systemically administered chemotherapy than the brain (Livshits et al., 2014). In contrast, there is a paucity of reports investigating the potential impact of chemotherapy on the SCN, which could drive circadian disruption throughout the body. Direct action of chemotherapeutics on SCN neurons is unlikely, as most chemotherapeutics do not readily cross the blood–brain barrier (Muldoon et al., 2007). For example, a common chemotherapeutic, paclitaxel (Weaver, 2014), shortens the length of a full oscillation (i.e., circadian period) of SCN clock protein rhythmicity when applied directly to a mouse SCN slice in vitro but not when paclitaxel is administered in vivo (Sullivan et al., 2022). Another chemotherapeutic, doxorubicin, administered in vivo does not disrupt SCN rhythmic activity measured via electrophysiology (Wang et al., 2024). Conversely, 5-fluorouracil readily crosses the blood–brain barrier (Han et al., 2008) and, when administered in vivo, damps rhythmic expression of SCN molecular clock genes (Terazono et al., 2008). This contradicting information regarding chemotherapy-induced effects on the SCN is inconclusive as to whether the principal clock itself is disrupted by chemotherapy, whether it be SCN neurons indirectly disrupted through neuroinflammation or directly through chemotherapeutics passing into the brain parenchyma.

The purpose of the present study was to determine the extent to which paclitaxel chemotherapy disrupts the SCN molecular clock and functional output. To do this, we administered paclitaxel or vehicle to female mice and measured the rhythmicity of molecular clock- and circadian-associated genes within the isolated SCN tissue. We also determined how paclitaxel influences SCN-dependent behavioral responses (voluntary wheel running) to timed light challenges via jet lag and phase response curve (PRC) paradigms.

## Materials and Methods

### Mice

Female, multiparous retired breeder C57BL/6 mice 6–10 months old (Charles River Laboratories) were used to approximate typical middle-aged women with breast cancer (Cronin et al., 2018). Mice were housed in temperature-controlled (22 ± 3°C), light-tight circadian cabinets with standard rodent chow and water available *ad libitum*. Cages included corn cob bedding and a cotton nestlet. All cabinets had white light with light intensity <1,000 lux. All mice were entrained to a 12 h light:12 h dark (12:12 L:D) cycle for 14 d prior to the experiment. Mice were group-housed (2–4 mice/cage) for the molecular SCN timekeeping experiment and singly housed for the running wheel experiments. Mice were disturbed infrequently, and cages were changed at random times under dim red light to minimize disruption to running rhythms. All experimental procedures were performed with prior approval from the Ohio State University Institutional Animal Care and Use Committee and based on standards listed in the National Institutes of Health Guide for the Care and Use of Laboratory Animals.

### Experimental Design

This study consisted of three experiments designed to determine the extent to which chemotherapy affects the SCN.

#### Experiment 1: molecular SCN timekeeping

**Figure 1.**
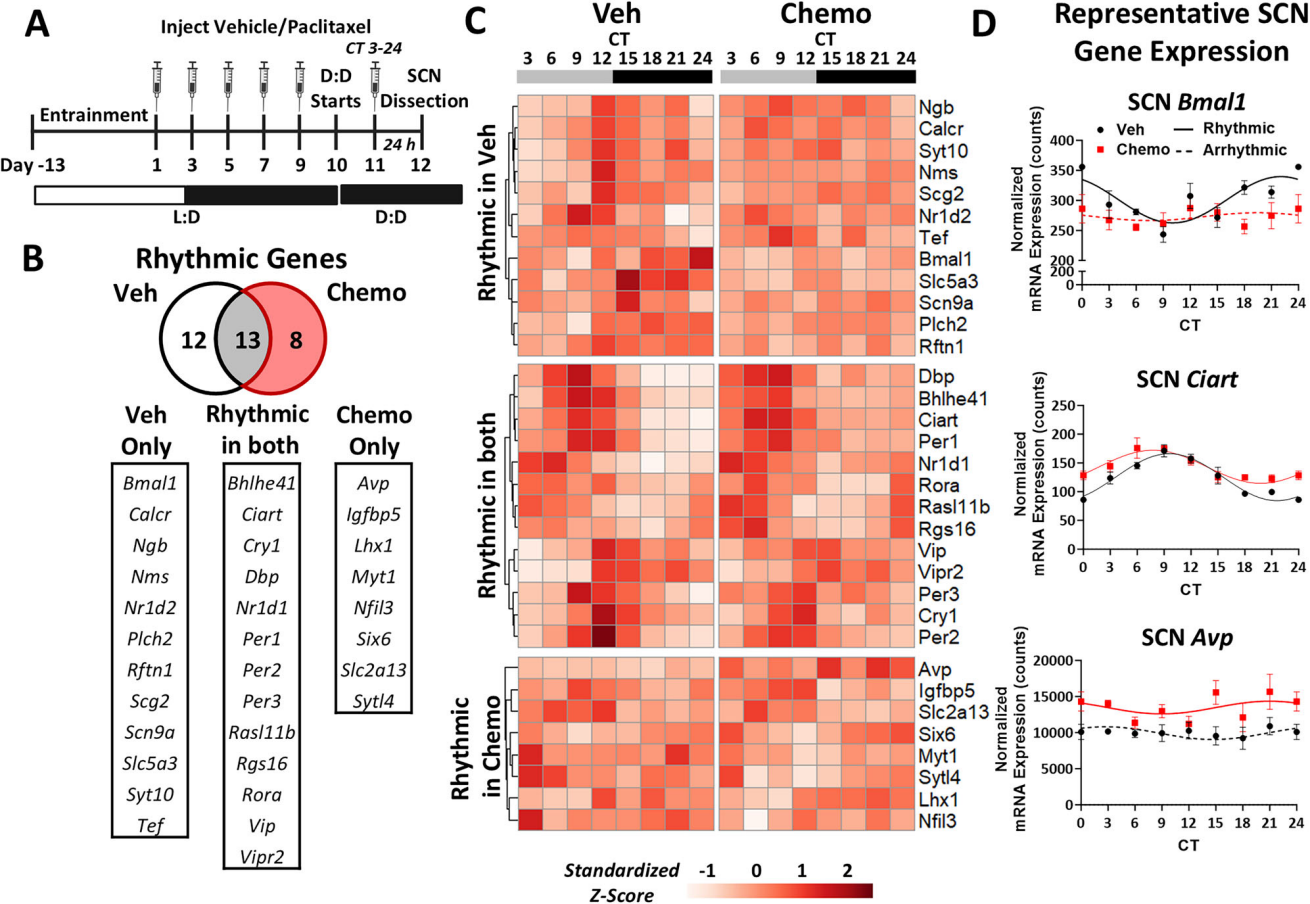
Chemotherapy disrupts rhythmic expression of molecular clock genes in the SCN. ***A***, Mice were given intraperitoneal injections of either paclitaxel or vehicle control every other day for six doses. Exactly 24 h after the last dose, the SCN tissue was microdissected for RNA analysis of circadian gene expression at one of the eight time points across a 24 h period. ***B***, Thirteen SCN genes were rhythmic in both chemotherapy- and vehicle-treated mice, but only 8 genes were uniquely rhythmic after chemotherapy; 12 genes were uniquely rhythmic after vehicle. ***C***, Standardized *z* scores of SCN gene expression in genes rhythmic only in Veh, rhythmic in both, and rhythmic only in Chemo. ***D***, Representative graphs of gene expression only rhythmic in Veh (*Bmal1*), both groups (*Ciart*), or only Chemo (*Avp*) with sine wave fit using least squares regression. Solid lines indicate significantly rhythmic expression (rain adj-*p* < 0.05), and dotted lines indicate arrhythmic expression (rain adj-*p* > 0.05). *N* = 4–5/group/time point.

To assess molecular clock and circadian-associated gene rhythm profiles within the SCN, the SCN tissue was collected from chemotherapy- and vehicle-treated mice (Fig. 1*A*). Group-housed mice were randomized into paclitaxel chemotherapy (“Chemo”) or vehicle (“Veh”) treatment groups and injected intraperitoneally six times over an 11 d period. Mice were then placed in constant darkness (D:D) 48 h before tissue collection to remove the entraining effects of light (Hatori et al., 2014; Hughes et al., 2017; Alzate-Correa et al., 2021). The last injection was timed to be 24 h prior to the designated SCN tissue collection time, every 3 h over 24 h at circadian time (CT) 3, 6, 9, 12, 15, 18, 21, and 24 (Veh *n* = 8/time point; Chemo *n* = 10/time point) to analyze rhythms in circadian gene expression. Given the precision of the timing for this experiment, four treatment-balanced cohorts of mice were run. All tissues were collected within 1 h of the designated CT. Gene expression analysis was conducted to assess rhythmicity of SCN transcripts.

#### Experiment 2: jet lag phase-advance and phase-delay paradigms

**Figure 2.**
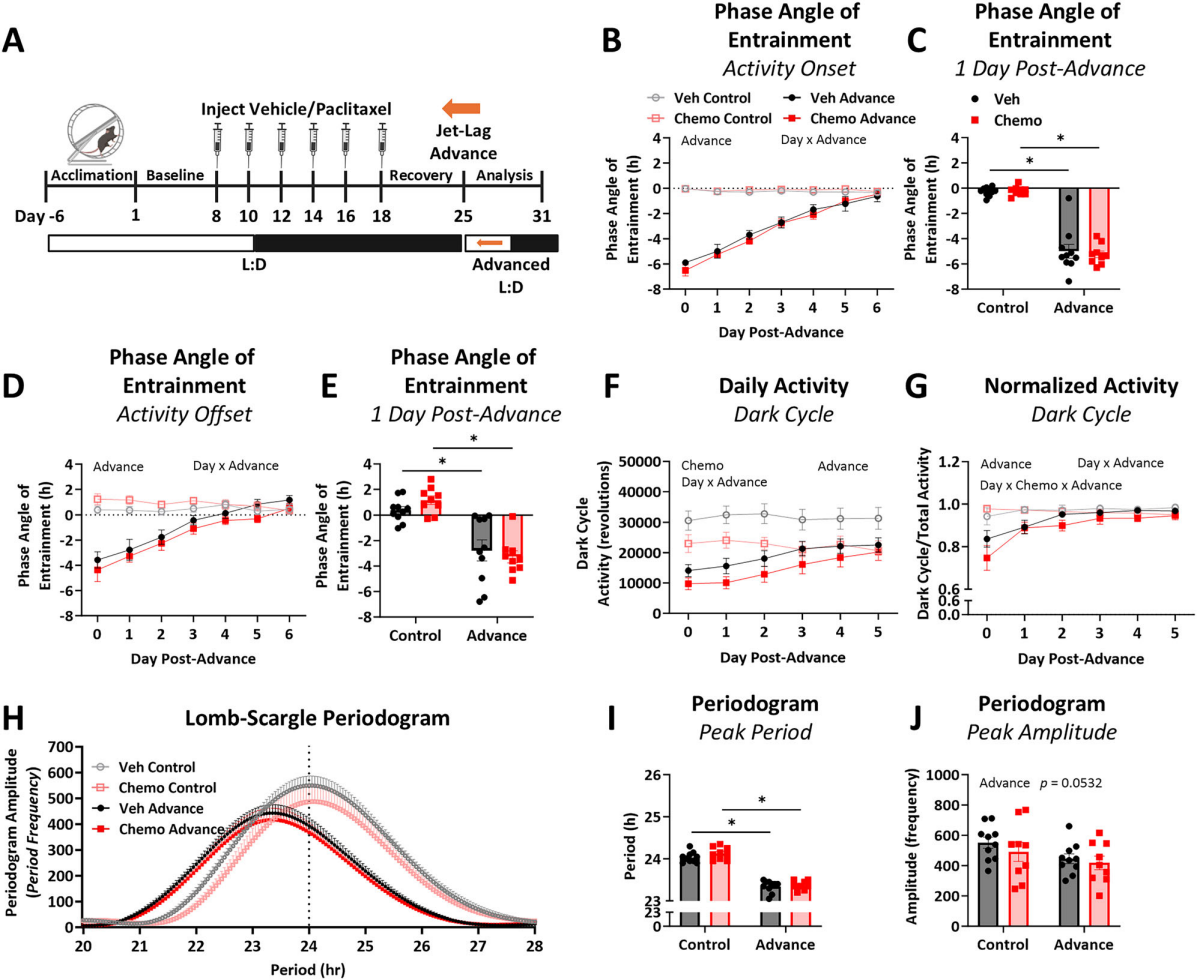
Chemotherapy does not alter re-entrainment to a 6 h phase-advance. ***A***, One week after injections, chemotherapy- and vehicle-treated mice were exposed to a 6 h phase-advance of their lights-on time, resulting in a short night followed by an advanced 12:12 L:D cycle. ***B***, The phase angle of entrainment for activity onset with a negative number indicating phase-delay relative to lights off. ***C***, Activity onset phase angle of entrainment at 1 d postadvance to determine chemotherapy-induced differences immediately after phase-advance. ***D***, The phase angle of entrainment for activity offset with a negative number indicating phase-delay relative to lights on. ***E***, Activity offset relative to lights on 1 d postadvance to determine chemotherapy-induced differences immediately after phase-advance. ***F***, Daily activity during the dark cycle, or active phase, after phase-advance as quantified by number of voluntary wheel running revolutions. Daily activity during the light cycle, or inactive phase, is quantified in Extended Data Figure 2-1*B*. ***G***, Dark cycle activity normalized to total activity (Extended Data Fig. 2-1*A*) as an indicator of circadian behavior re-entrainment to phase-advance, with a number <1.0 indicating wheel running occurred during the light cycle. ***H***, Lomb–Scargle periodogram of the week following the phase-advance shift. ***I***, Peak period from Lomb–Scargle periodogram as an indicator of the strongest circadian period during the analyzed time. ***J***, Peak amplitude from Lomb–Scargle periodogram acts as an indicator of circadian strength through plotting the frequency at which the peak period occurred. *N* = 9–10/group. Two-way ANOVA main or interaction effects listed on graphs, *p* < 0.05. * Fisher's LSD test between indicated groups, *p* < 0.05.

**Figure 3.**
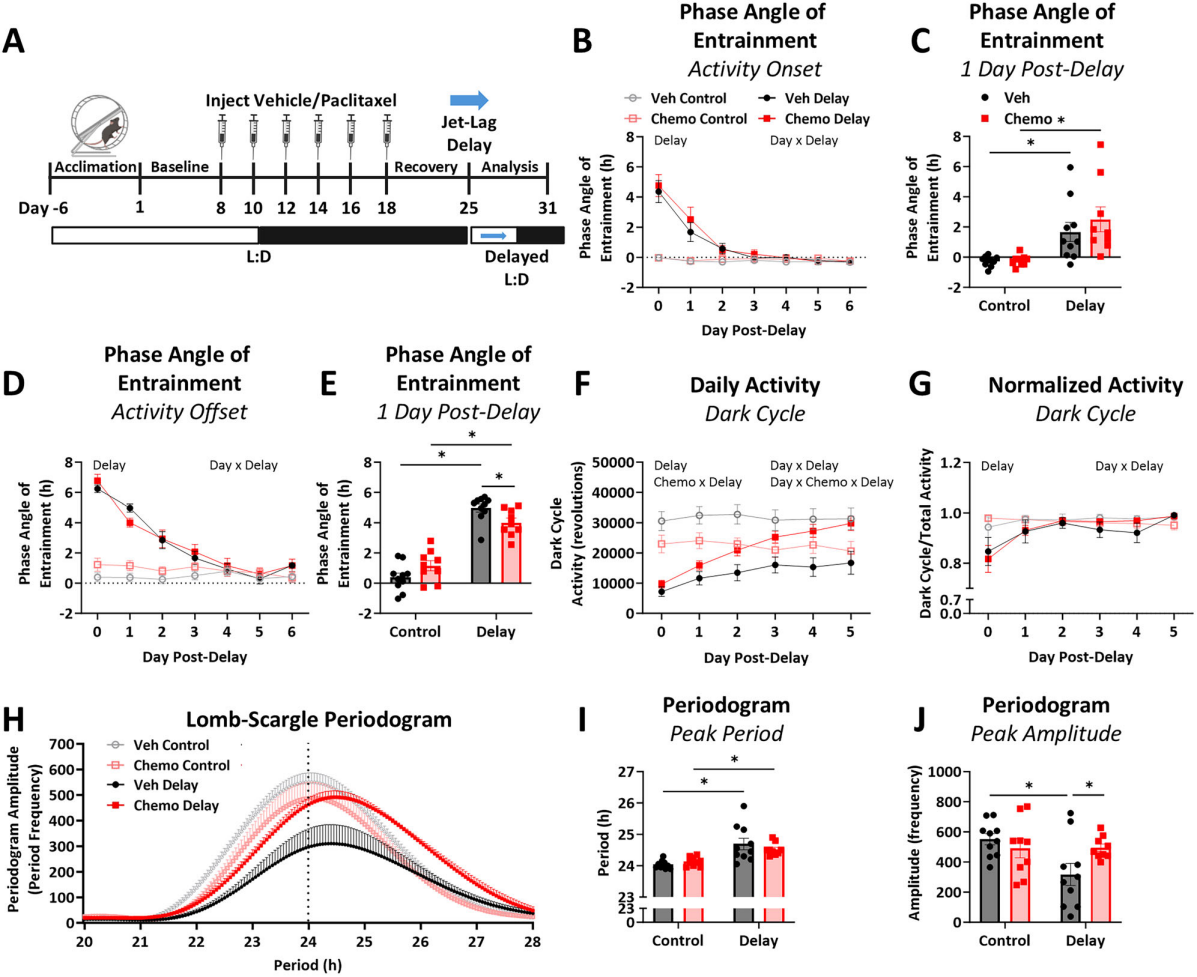
Chemotherapy enhances SCN-dependent re-entrainment to a 6 h phase-delay. ***A***, After 1 week of recovery from injections, chemotherapy- and vehicle-treated mice were exposed to a 6 h phase-delay shift by delaying the offset of lights, resulting in a long day followed by a delayed 12:12 L:D cycle. ***B***, The phase angle of entrainment for activity onset with a positive number indicating phase-advance relative to lights off. ***C***, Activity onset phase angle of entrainment at 1 d post phase-delay to determine chemotherapy-induced differences immediately after phase-delay shift. ***D***, The phase angle of entrainment for activity offset with a positive number indicating phase-shift advance relative to lights on. ***E***, Activity offset relative to lights off 1 d postshift to determine chemotherapy-induced differences immediately after phase-delay. ***F***, Daily activity during the dark cycle, or active phase, after phase-delay as quantified by the number of voluntary wheel running revolutions. Daily activity during the light cycle, or inactive phase, is quantified in Extended Data Figure 2-1*D*. ***G***, Dark cycle activity normalized to total activity (Extended Data Fig. 2-1*C*) as an indicator of circadian behavior re-entrainment to phase-delay, with a number <1.0 indicating wheel running occurred during the light cycle. ***H***, Lomb–Scargle periodogram of the week following phase-delay shift. ***I***, Peak period from Lomb–Scargle periodogram as an indicator of the strongest circadian period during the analyzed time. ***J***, Peak amplitude from Lomb–Scargle periodogram acts as an indicator of circadian strength through plotting the frequency at which the peak period occurred. *N* = 9–10/group. Two-way ANOVA main or interaction effects listed on graph, *p* < 0.05. * Fisher’s LSD test between indicated groups, *p* < 0.05.

To assess the ability of the SCN to adjust to a new photoperiod, we used phase-advance (Fig. 2*A*) and phase-delay (Fig. 3*A*) jet lag paradigms. Mice were allowed 7 d to acclimate to their running wheels, and then baseline voluntary wheel running was recorded for the next 7 d. Mice were treated with paclitaxel or vehicle as described above. Following 7 d of recovery, both treatment groups were challenged with either **(1)** a 6 h phase-advanced light cycle (i.e., 12:12 L:D, lights turn on 6 h earlier; Veh *n* = 10; Chemo *n* = 9), **(2)** a 6 h phase-delayed light cycle (i.e., 12:12 L:D, lights turn off 6 h later; Veh *n* = 10; Chemo *n* = 9), or **(3)** no change to the photoperiod (Veh *n* = 10; Chemo *n* = 9). For phase-advance, preshift lights came on at ZT1, and postshift lights came on at ZT19, resulting in a short night. For phase-delay, preshift lights turned off ZT13, but postshift lights turned off ZT19, resulting in a long day. Day 0 of phase-advance or phase-delay was defined as beginning when the new L:D created deviation from the previous cycle (ZT19 for both phase-advance and phase-delay). Day 1 postshift was defined as the first fully adjusted L:D period following the phase-advance or phase-delay (Yamazaki et al., 2000). Wheel running responses after this challenge were recorded for an additional 7 d. The phase angle of entrainment (activity onsets and offsets relative to lights on and off, respectively), light and dark cycle wheel revolutions, and Lomb–Scargle periodogram after phase-advance or phase-delay were compared between treatments.

10.1523/ENEURO.0061-25.2025.f2-1 Figure 2-1 **Phase-advance and -delay differentially influence total wheel running activity after chemotherapy. (A)** Number of daily wheel revolutions acting as an indicator of total activity following phase-advance shift, or relative control period. **(B)** Number of wheel revolutions that occurred during the light cycle after phase-advance, indicating daily activity during the relative inactive phase. **(C)** Number of daily wheel revolutions acting as an indicator of total activity following the phase-delay shift, or relative control period. **(D)** Number of wheel revolutions that occurred during the light cycle after phase-delay, indicating daily activity during the relative inactive phase. N = 9-10/group. Two-way ANOVA main or interaction effects listed on graphs, *p* < 0.05. Download Figure 2-1, TIF file.

#### Experiment 3: PRC

**Figure 4.**
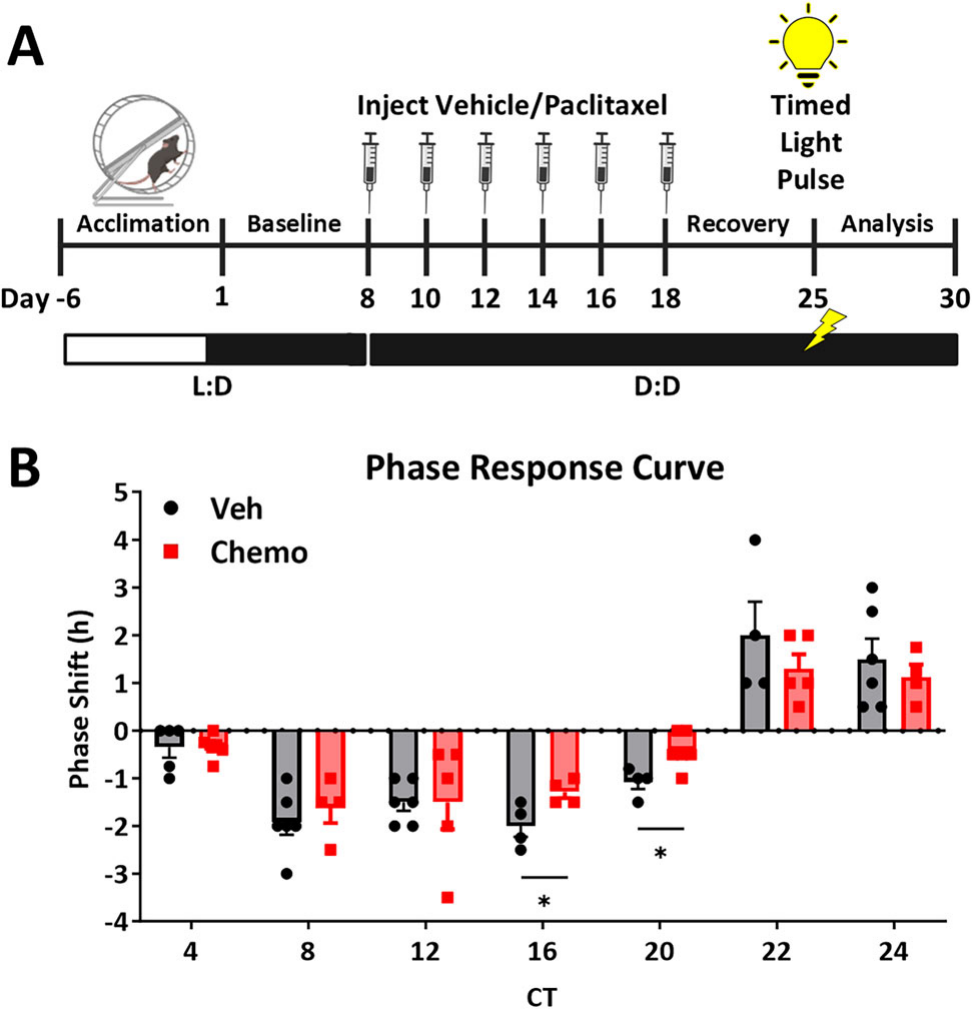
Chemotherapy blunts light pulse-induced phase-shift delay during subjective night. ***A***, Following entrainment to a 12:12 L:D cycle, mice were housed in constant darkness (D:D) beginning the first day of chemotherapy or vehicle injections and remained in D:D for the remainder of the experiment. Five to seven days after the last injection, mice were exposed to a 30 min pulse of light at one of seven CTs. ***B***, The light-induced phase-shift delay (negative number) or advance (positive number) 4 d after the light pulse. *N* = 4–6/group/time point. * Student's *t* test, *p* < 0.05.

To assess the ability of the primary clock to entrain to light pulses timed at specific points in the circadian day, we generated a PRC (Fig. 4*A*). Baseline wheel running was recorded for 7 d as described above then mice were put into D:D concurrent with their first paclitaxel or vehicle injection and remained in D:D for the rest of the experiment. Mice were given 5–7 d after the injection paradigm to recover, and CT12 was predicted by drawing a best-fit line along activity onsets during this post-treatment recovery period (Daan and Pittendrigh, 1976). Mice then received a single 30 min light pulse (90–110 lux, white, fluorescent bulb 3,500 K, 32 W) at one of seven different CTs (4, 8, 12, 16, 20, 22, 24) based on their predicted CT12. Subsequent running wheel rhythms were recorded for 5 d. To calculate the phase response to each timed light pulse, a best-fit line was created using activity onsets after injections (recovery phase; 5–7 d) as well as using activity onsets following the light pulse (experimental phase; 5 d). The PRC phase-shift was calculated as the difference to the closest 15 min increment between the recovery phase and the experimental phase with a negative value indicating phase-delay and a positive value indicating phase-advance. A PRC was obtained by plotting these phase-shifts across time of the light pulses. The magnitude of the phase-advance/delay was compared between treatments.

### Chemotherapy treatment

Paclitaxel chemotherapy (LC Laboratories, catalog #P-9600, and Tocris Bioscience, catalog #1097) was dissolved in Cremophor EL:PBS solution (Millipore-Sigma, catalog #238470-1SET). Chemotherapy (30 mg/kg in 100 μl, i.p.), or vehicle was injected every other day at variable times within the light phase for a total of six doses. Variable injection times were used to prevent entrainment to injection timing. Mice were pseudorandomized into chemotherapy or vehicle groups based on equivalent initial body mass. Body mass was measured every other day and mice were killed and excluded from the study if they lost >20% of their baseline body mass. Due to these exclusion criteria, one chemotherapy mouse was killed in Experiment 1 from each of the following timepoint groups: CT3, CT12, and CT18. Additionally, two chemotherapy mice were killed from the CT15 group. In Experiment 2, two chemotherapy mice were killed, one in each of the advance and delay groups.

### SCN isolation and processing

For the gene expression experiment, mice were rapidly decapitated under photobiologically inert red light; the brain was carefully dissected to ensure integrity of the optic chiasm and placed in chilled Hanks Balanced Salt Solution (HBSS; Thermo Fisher Scientific, catalog #14-175-145) for 30 s. The brain was affixed to a vibratome (Leica Biosystems, catalog #VT1000 S) as previously described (Obrietan et al., 1999). Briefly, the olfactory bulbs and cerebellum were removed with a razor blade, and the brain was secured on the vibratome stage with cyanoacrylate, submerged in chilled HBSS, and sectioned coronally until the optic chiasm was reached. Six hundred micrometer sections were then taken and observed under a dissection microscope (Olympus, catalog #SZ30) for identification of the SCN. Once identified, the SCN was microdissected to a ∼1.0 × 1.0 mm × 600 µm square using microscissors (World Precision Instruments, catalog #14003), placed into RNALater preservative (Invitrogen, catalog #AM7020), rapidly frozen on dry ice, and stored at −80°C until RNA was extracted using the RNeasy Mini Kit (Qiagen, catalog #74106) following manufacturer’s instructions. Due to experimental restraints of keeping a consistent 24 h between the final injection and tissue collection for all time points, the SCN tissue was collected over one circadian cycle (24 h) with high temporal resolution (sampling interval 3 h; Hughes et al., 2017). Additionally, two SCN samples from different mice in the same treatment group (Veh, Chemo) and CT (3, 6, 9, 12, 15, 18, 21, 24) were randomly pooled together across cohorts to reduce variability in gene expression resulting in *n* = 4/group for Veh and *n* = 5/group for Chemo. RNA concentrations were measured using a NanoDrop spectrophotometer (Thermo Fisher Scientific, catalog #ND-ONE-W) with A260/A280 ratios ∼1.8–2.0.

### SCN gene expression analysis

RNA samples were run on a custom nCounter panel (NanoString Technologies) to assess expression of four housekeeping and 50 circadian-associated genes between chemotherapy- and vehicle-treated mice over the 24 h following treatment using the OSU Comprehensive Cancer Center Genomics Shared Resource Facility. Raw mRNA counts were quality controlled and normalized using the nCounter Analysis Software (NanoString Technologies). One Chemo CT18 sample was excluded due to significantly lower housekeeping gene expression. This resulted in *n* = 5/group for Chemo CT3, 6, 9, 12, 15, 21, and 24; *n* = 4/group for Chemo CT18; and *n* = 4/group for all Veh CTs. Rhythmic gene expression was determined by the R package rain (v1.38) on normalized mRNA counts using the independent analysis method and Benjamini–Hochberg false discovery rate correction (Thaben and Westermark, 2014). Differences in midline-estimating statistic of rhythm (MESOR), amplitude, and phase of genes that were rhythmic in both Veh and Chemo groups were determined by R package circaCompare (v0.2.0; Parsons et al., 2020). Heatmaps of rhythmically expressed genes were made with R package pheatmap (v1.0.12) with each box representing the standardized *z* score of normalized mRNA counts from the indicated time point within Veh or Chemo mice. To further visualize chemotherapy-induced changes to rhythmic gene expression, we plotted normalized mRNA counts with a sine wave fit using least squares regression with a 24 h period in GraphPad Prism (v10.0.2; GraphPad Software).

### Wheel running

Voluntary wheel running revolutions were continuously recorded using a probe attached to an in-cage running wheel (4.5 in dia) and the VitalView software (STARR Life Sciences). The VitalView 6 software was used for Experiment 2, and the VitalView 1.5 software was used for Experiment 3. Wheel running data were analyzed in 5 min bins with the ClockLab Analysis 6 Software (Actimetrics). Activity onsets and offsets were automatically calculated by ClockLab and manually adjusted if automatic designations did not meet criteria for onset or offset. The activity onset was defined as sustained activity for 30 min preceding at least 80% of total activity for that 24 h period. Activity offset was defined as inactivity for 30 min after at least 80% of total activity for that 24 h period. Within the experimental period, analysis subphases include the postadvance or postdelay period (5 d after advance or delay) and the phase-shift response to timed light pulses in PRC (5 d after light pulses).

### Statistical analyses

In Experiment 1, gene expression was considered rhythmic when the rain adjusted *p* < 0.05. Chemotherapy-induced changes to circadian characteristics of genes that were rhythmic in both Veh and Chemo groups were considered significant when circaCompare *p* < 0.05. All other statistical comparisons were performed using GraphPad Prism (v10.0.2; GraphPad Software). To determine chemotherapy-induced changes to expression of genes that were either rhythmic in only one group or not rhythmic in either group, genes were analyzed by two-way ANOVA to determine significant main effects of treatment (Veh, Chemo) and time (CT3-24), as well as interaction effects (treatment × time). Only main effects of chemotherapy are reported. For Experiment 2, chemotherapy-induced changes to the phase angle of entrainment and wheel running activity were analyzed by three-way ANOVA with repeated measures to determine main effects of treatment (Veh, Chemo), condition (control, advance or delay), and time (day postadvance or postdelay; repeated measure), as well as interaction effects. Significant main effects of day were identified in all repeated-measure analyses and are not reported on the graphs but are detailed in text. All other significant main or interaction effects are labeled on the relevant graphs. The phase angle of entrainment immediately after phase-advance or phase-delay, as well as periodogram peak period and amplitude, was analyzed by two-way ANOVA to determine main effects of treatment (Veh, Chemo) and condition (control, advance or delay), as well as interaction effects (treatment × condition). In all two-way ANOVA analyses, significant main or interaction effects were followed by multiple-comparison testing with Fisher's LSD test. For Experiment 3, Student's *t* test compared light-induced phase-shifts at timed light pulse between Veh and Chemo groups when variance was normal, or Mann–Whitney *U* tests were used when variance was nonparametric. In all ANOVA, Fisher's LSD test, and *t* test analyses, significance was determined as *p* < 0.05 and specific *p* values are reported unless they were <0.0001.

## Results

### Chemotherapy disrupts rhythmic expression of molecular clock genes in the SCN

Rhythmicity of 50 circadian-associated SCN transcripts was used as a measure of molecular clock integrity in female mice treated with chemotherapy or vehicle (Fig. 1*A*). Statistically significant 24 h rhythms were detected in 13 genes regardless of treatment, while vehicle-treated mice had rhythmic expression of 12 unique transcripts and chemotherapy-treated mice only had 8 unique rhythmically expressed transcripts (Fig. 1*B*). Heatmaps show the mean standardized *z* score for each CT tissue was collected (Fig. 1*C*). Representative graphs (Fig. 1*D*) show that chemotherapy abolished rhythmic expression of SCN *Bmal1* relative to vehicle control (top; Veh rain adj-*p* < 0.0001), SCN *Ciart* was rhythmically expressed after both chemotherapy or vehicle exposure; however, *Ciart* was out-of-phase between groups (middle; Veh rain adj-*p* < 0.0001; Chemo rain adj-*p* < 0.0001), and SCN *Avp* was rhythmic only with chemotherapy treatment (bottom; Chemo rain adj-*p* = 0.027).

### Chemotherapy alters both rhythmic and arrhythmic circadian gene expression

Within genes that were rhythmically expressed in the SCN of both chemotherapy- and vehicle-treated mice, chemotherapy caused alterations in oscillation characteristics (Table 1). For example, chemotherapy increased the MESOR of *Ciart*, *Dbp*, and *Nr1d1* (*Ciart*, *p* = 0.0003; *Dbp*, *p* = 0.0002; *Nr1d1*, *p* = 0.002), indicating an overall increased expression relative to vehicle-treated mice across time. Chemotherapy also decreased the expression amplitude of *Dbp*, *Per1*, *Per2*, and *Per3* (*Dbp*, *p* = 0.001; *Per1*, *p* = 0.038; *Per2*, *p* = 0.009; *Per3*, *p* = 0.004) indicating a damped rhythm compared with vehicle controls. Finally, chemotherapy caused a phase-shift in SCN *Bhlhe41*, *Ciart*, *Dbp*, and *Per2* rhythms (*Bhlhe41*, *p* = 0.003; *Ciart*, *p* = 0.018; *Dbp*, *p* = 0.012; *Per2*, *p* = 0.044).

**Table 1.** Chemotherapy alters circadian characteristics of rhythmic SCN gene expression

Gene	Vehicle			Chemotherapy			Chemotherapy-induced difference					
	MESOR	Amplitude	Peak (CT)	MESOR	Amplitude	Peak (CT)	MESOR Difference	*p* value	Amplitude Difference	*p* value	Phase Shift (h)	*p* value
*Bhlhe41*	1,119.7	127.23	9.96	1,119.27	74.71	6.68	−0.43	0.982	−52.53	0.0501	3.27 *	0.003
*Ciart*	126.03	39.57	9.5	143.6	28.48	7.7	17.57*	0.0003	−11.08	0.094	1.80*	0.018
*Cry1*	191.01	27.25	13.43	185.54	17.66	10.95	−5.47	0.317	−9.6	0.213	2.47	0.077
*Dbp*	1,194.57	404.51	8.61	1,327.48	242.87	7.05	132.91*	0.0002	−161.65*	0.001	1.56*	0.012
*Nr1d1*	131.02	34.01	5.93	145.89	21.8	4.28	14.88*	0.002	−12.21	0.062	1.65	0.077
*Per1*	327.11	68.92	10.05	339.91	40.76	8.19	12.8	0.178	−28.17*	0.038	1.87	0.065
*Per2*	459.91	139.7	11.63	460.77	85.38	10.12	0.86	0.952	−54.32*	0.009	1.51*	0.044
*Per3*	233.49	38.47	11.72	240.85	14.64	11.35	7.36	0.198	−23.83*	0.004	0.36	0.813
*Rasl11b*	101.94	27.37	4.16	107.37	35.76	3.26	5.43	0.393	8.39	0.351	0.9	0.427
*Rgs16*	493.97	86.15	4.23	535.8	165.35	3.79	41.83	0.228	79.21	0.109	0.44	0.442
*Rora*	1,141.98	97.59	6.88	1,127.91	133.98	5.48	−14.07	0.694	36.4	0.475	1.4	0.427
*Vip*	784.55	316.55	14.58	850.96	169.81	13.31	66.41	0.349	−146.73	0.144	1.27	0.473
*Vipr2*	258.9	98.19	16.97	261.8	67.62	16.63	2.9	0.865	−30.58	0.208	0.35	0.760

Estimated values using circaCompare for MESOR, amplitude, and peak in SCN genes rhythmic in both vehicle- and chemotherapy-treated mice. Chemotherapy-induced differences determined as circaCompare *p* < 0.05. Chemotherapy-induced differences of genes that are only rhythmic in one group or neither group are also determined (Extended Data Table 1-1). CT, circadian time. *n* = 4–5/group/time point.

10.1523/ENEURO.0061-25.2025.t1-1 Table 1-1 Chemotherapy alters overall expression of circadian-associated genes regardless of rhythmicity. Download Table 1-1, DOC file.

Chemotherapy further altered expression of genes that were either not rhythmically expressed by either group or only rhythmic in one group but not the other. To determine if there were still chemotherapy-induced changes to these transcripts regardless of circadian rhythmicity, main effects of chemotherapy were determined (Extended Data Table 1-1).

SCN *Bmal1*, *Rftn1*, and *Slc5a3* genes were all only rhythmically expressed after vehicle treatment (Veh, rain adj-*p* < 0.001) and had decreased mean expression with chemotherapy (*Bmal1*, Chemo, *F*_(1, 55)_ = 10.92; *p* = 0.0017; *Rftn1*, Chemo, *F*_(1, 55)_ = 4.369; *p* = 0.0412; *Slc5a3*, Chemo, *F*_(1, 55)_ = 22.33; *p* < 0.0001). In contrast, SCN expression of *Avp* was rhythmic with chemotherapy (Chemo, rain adj-*p* = 0.027) and was the only transcript with increased mean expression following chemotherapy treatment (Chemo, *F*_(1, 55)_ = 26.22; *p* < 0.0001). All other SCN transcripts that were only rhythmic with chemotherapy treatment (*Myt1*, *Nfil3*, *Sytl4*; Chemo, rain adj-*p* < 0.05) were all decreased compared with vehicle (*Myt1*, Chemo, *F*_(1, 55)_ = 6.085; *p* = 0.0168; *Nfil3*, Chemo, *F*_(1, 55)_ = 6.654; *p* = 0.0126; *Sytl4*, Chemo, *F*_(1, 55)_ = 7.075; *p* = 0.0102). Finally, SCN expression of *Clock*, *Csnk2a1*, *Epha6*, *Npas2*, and *Trp53i11* was nonrhythmic regardless of treatment but was overall decreased by chemotherapy compared with vehicle controls (*Clock*, Chemo, *F*_(1, 55)_ = 12.97; *p* = 0.0007; *Csnk2a1*, Chemo, *F*_(1, 55)_ = 7.033; *p* = 0.0104; *Epha6*, Chemo, *F*_1, 55_ = 9.897; *p* = 0.0027; *Npas2*, Chemo, *F*_(1, 55)_ = 8.694; *p* = 0.0047; *Trp53i11*, Chemo, *F*_(1, 55)_ = 4.207; *p* = 0.045).

### Chemotherapy does not alter re-entrainment to a 6 h phase-advance

To determine the extent to which chemotherapy impacts SCN function, we assessed the rate of re-entrainment and stability of circadian adaptation to a 6 h phase-advance in the L:D cycle 7 d after chemotherapy or vehicle treatment (Fig. 2*A*). As expected, 6 h phase-advance triggered a delay in the phase angle of entrainment for the onset of running wheel activity regardless of treatment (Fig. 2*B*; advance, *F*_(1, 34)_ = 201.5; *p* < 0.0001), which recovered over time (day, *F*_(3.959, 134.6)_ = 94.34; *p* < 0.0001; interaction day × advance, *F*_(6, 204)_ = 103.3; *p* < 0.0001). To determine if chemotherapy affected adaptation to the phase-advance immediately following the challenge, we isolated onset of activity the day after the phase-advance (Fig. 2*C*) and found that wheel running onset of activity was delayed relative to lights on regardless of treatment (advance, *F*_(1, 34)_ = 222.7; *p* < 0.0001; Veh control–Veh advance, *p* < 0.0001; Chemo control–Chemo advance, *p* < 0.0001). The phase angle of entrainment for offset of activity can similarly indicate adaptation to circadian challenges; thus offsets of wheel running activity relative to lights on were also assessed (Meijer and de Vries, 1995). As with activity onset, phase-advance triggered a delay in activity offset relative to lights on (Fig. 2*D*; advance, *F*_(1, 34)_ = 35.85; *p* < 0.0001) which recovered over time with no influence of chemotherapy (day, *F*_(3.310, 112.5)_ = 27.07; *p* < 0.0001; interaction day × advance, *F*_(6, 204)_ = 33.82; *p* < 0.0001) and was also not affected by treatment the day after phase-advance (Fig. 2*E*; advance, *F*_(1, 34)_ = 49.44; *p* < 0.0001; Veh control–Veh advance, *p* = 0.0002; Chemo control–Chemo advance, *p* < 0.0001).

In line with previous reports (Sullivan et al., 2022), paclitaxel caused fatigue (i.e., decreased voluntary running wheel revolutions) during the dark cycle or active phase (Fig. 2*F*; Chemo, *F*_(1, 34)_ = 6.490; *p* = 0.016), which was similarly reflected in total daily activity (Extended Data Fig. 2-1*A*; Chemo, *F*_(1, 34)_ = 7.413; *p* = 0.01). Phase-advance independently contributed to fatigue during the dark cycle (Fig. 2*F*; advance, *F*_(1, 34)_ = 15.48; *p* = 0.0004) and total daily activity (Extended Data Fig. 2-1*A*; advance, *F*_(1, 34)_ = 5.943; *p* = 0.02). This advance-induced fatigue recovered over time in both the dark cycle activity (Fig. 2*F*; day, *F*_(2.495, 82.34)_ = 6.705; *p* = 0.0009; interaction day × advance, *F*_(5, 165)_ = 10.99; *p* < 0.0001) and total daily activity (Extended Data Fig. 2-1*A*; day, *F*_(3.359, 110.8)_ = 3.832; *p* = 0.009; interaction day × advance, *F*_(5, 166)_ = 4.003; *p* = 0.002). As expected, phase-advance increased activity during the light cycle (Extended Data Fig. 2-1*B*; advance, *F*_(1, 34)_ = 15.94; *p* = 0.0003) regardless of treatment. This light cycle activity steadily recovered over time regardless of treatment (day, *F*_(1.487, 49.08)_ = 18.71; *p* < 0.0001; interaction day × advance, *F*_(5, 165)_ = 17.93; *p* < 0.0001). As a measure of activity re-entrainment that accounts for chemotherapy- or advance-induced fatigue, we normalized dark cycle running activity to total activity, with values <1.0 indicating activity occurred during the lights-on period when mice should typically be inactive. As expected, the phase-advance decreased the proportion of wheel running activity during the dark cycle (Fig. 2*G*; advance, *F*_(1, 34)_ = 9.881; *p* = 0.004), while the proportion of activity during the dark cycle increased over time after phase-advance (day, *F*_(1.767, 60.08)_ = 15.84; *p* < 0.0001; interaction day × advance, *F*_(5, 170)_ = 13.62; *p* < 0.0001). Notably, this recovery was modestly slower in mice treated with chemotherapy (interaction day × advance × chemo, *F*_(5, 170)_ = 2.274; *p* = 0.049).

A Lomb–Scargle periodogram illustrated the primary circadian period and circadian strength of wheel running activity 5 d following the phase-advance after chemotherapy treatment (Fig. 2*H*). Phase-advance shortened the circadian period (Fig. 2*I*; advance, *F*_(1, 34)_ = 290.8; *p* < 0.0001; Veh control–Veh advance, *p* < 0.0001; Chemo control–Chemo advance, *p* < 0.0001) with no influence of prior chemotherapy treatment. Phase-advance caused a trending decrease in periodogram amplitude, an indicator of circadian stability, regardless of chemotherapy treatment (Fig. 2*J*; advance, *F*_(1, 34)_ = 4.013; *p* = 0.053).

### Chemotherapy enhances SCN-dependent re-entrainment to a 6 h phase-delay

A separate cohort of mice were exposed to a 6 h phase-delay in the L:D cycle 7 d after chemotherapy or vehicle treatment (Fig. 3*A*). Phase-delay triggered an advance in the activity onset phase angle of entrainment, which rapidly recovered regardless of chemotherapy treatment (Fig. 3*B*; day, *F*_(2.161, 73.48)_ = 51.86; *p* < 0.0001; delay, *F*_(1, 34)_ = 36.38; *p* < 0.0001; interaction day × delay, *F*_(6, 204)_ = 45.63; *p* < 0.0001). Activity onset was not different with chemotherapy when looking acutely during the first day after phase-delay (Fig. 3*C*; delay, *F*_(1, 34)_ = 20.35; *p* < 0.0001; Veh control–Veh delay, *p* = 0.01; Chemo control–Chemo delay, *p* = 0.0009). There was also no influence of chemotherapy on adaptation of activity offset to phase-delay over time (Fig. 3*D*; day, *F*_(4.703, 159.9)_ = 76.20; *p* < 0.001; delay, *F*_(1, 34)_ = 50.90; *p* < 0.0001; interaction day × delay, *F*_(6, 204)_ = 64.81; *p* < 0.0001). However, in the first day after the phase-delay occurred (Fig. 3*E*), while there was an overall advance of activity offset (delay, *F*_(1, 34)_ = 158.0; *p* < 0.0001; Veh control–Veh delay, *p* < 0.0001; Chemo control–Chemo delay, *p* < 0.0001), chemotherapy-treated mice more quickly adapted their activity offset compared with vehicle-treated mice (interaction delay × chemo, *F*_(1, 34)_ = 8.688; *p* = 0.006; Veh delay–Chemo delay, *p* = 0.028).

Similar to the phase-advance paradigm, phase-delay caused fatigue during the dark cycle (Fig. 3*F*; delay, *F*_(1, 34)_ = 14.23; *p* = 0.0006) that is also reflected in daily total activity (Extended Data Fig. 2-1*C*; delay, *F*_(1, 34)_ = 7.247; p = 0.01). This delay-induced fatigue recovered over time in both the dark cycle activity (Fig. 3*F*; day, *F*_(2.460, 81.17)_ = 13.86; *p* < 0.0001; interaction day × delay, *F*_(5, 165)_ = 17.82; *p* < 0.0001) and total daily activity (Extended Data Fig. 2-1*C*; day, *F*_(2.570, 84.80)_ = 6.912; *p* = 0.0006; interaction day × delay, *F*_(5, 166)_ = 7.339; *p* < 0.0001). Also similar to the phase-advance paradigm, phase-delay caused an increase in activity during the light cycle (Extended Data Fig. 2-1*D*; delay, *F*_(1, 34)_ = 17.37; *p* = 0.0002), but this recovered over time with no influence of chemotherapy (Extended Data Fig. 2-1*D*; day, *F*_(1.562, 51.54)_ = 26.33; *p* < 0.0001; day × delay, *F*_(5, 165)_ = 25.65; *p* < 0.0001). Notably, chemotherapy-treated mice recovered from delay-induced fatigue during the dark cycle more rapidly (Fig. 3*F*; interaction chemo × delay, *F*_(1, 34)_ = 11.49; *p* = 0.002; interaction day × chemo × delay, *F*_(5, 165)_ = 3.584; *p* = 0.004). We again normalized dark cycle running activity to total activity, with values <1.0 indicating activity occurred during the lights on period when mice should typically be inactive. As expected, phase-delay decreased the proportion of wheel running activity during the dark cycle, which recovered over time (Fig. 3*G*; day, *F*_(1.875, 63.76)_ = 8.602; *p* = 0.0006; interaction day × delay, *F*_(5, 170)_ = 7.085; *p* < 0.0001).

Based on a Lomb–Scargle periodogram (Fig. 3*H*), phase-delay lengthened the circadian period regardless of treatment (Fig. 3*I*; delay, *F*_(1, 34)_ = 24.50; *p* < 0.0001; Veh control–Veh delay, *p* < 0.0001; Chemo control–Chemo delay, *p* = 0.015). However, only vehicle-treated mice had decreased circadian stability with phase-delay exposure, indicated by periodogram amplitude (Fig. 3*J*; delay, *F*_(1, 34)_ = 4.611; *p* = 0.039; interaction delay × chemo, *F*_(1, 34)_ = 4.886; *p* = 0.034; Veh control–Veh delay, *p* = 0.003; Veh delay–Chemo delay, *p* = 0.026).

### Chemotherapy blunts light pulse-induced phase-shift delay during subjective night

To assess the ability of the SCN to rapidly respond to a brief environmental light stimulus, ∼1 week after injections, multiple cohorts of mice were exposed to a light pulse at a single CT, and a phase response curve was generated (Fig. 4*A*). Chemotherapy blunted light-induced phase-shift delays at CT16 (*t*_(6)_, *p* = 0.034) and CT20 (*t*_(8)_, *p* = 0.019), indicating a decreased ability of the SCN to appropriately respond to delay-inducing light cues uniquely during subjective night (Fig. 4*B*). There were no effects of chemotherapy treatment on light-induced phase advances.

## Discussion

The present study examined how paclitaxel chemotherapy disrupts the SCN molecular clock and alters SCN-dependent behavioral adaptations to circadian challenges in female mice. Chemotherapy-induced circadian disruption is reported both clinically and in mouse models (Amidi and Wu, 2022; Tapp et al., 2025); however, involvement of the principal clock in this disruption remains inconclusive. Here, the results indicate that paclitaxel chemotherapy-induced disruption indeed occurs at the level of the SCN, which may mediate or synergize with previously reported downstream peripheral clock disruption (Buijs et al., 2013; Ramkisoensing and Meijer, 2015). SCN dysfunction may contribute to the observed associations between circadian disruption, decreased quality of life, and increased mortality in clinical cancer populations (Innominato et al., 2009; Milanti et al., 2021).

We first determined how paclitaxel impacts gene expression relevant to SCN molecular clock function and regulation (Shearman et al., 2000; Ko and Takahashi, 2006). To our knowledge, this is the first extensive characterization of the SCN molecular clock after chemotherapy exposure. The SCN maintains a robust circadian rhythm that is quite resilient to perturbations (Butler and Silver, 2009). Here, chemotherapy robustly altered molecular clock gene expression of the SCN. We not only found evidence of disruption through ablated rhythmic expression of the key molecular clock gene, *Bmal1*, but also through chemotherapy-induced changes to various other characteristics of rhythmicity (e.g., phase). Indeed, circadian disruption is defined as any disturbance to the typical 24 h rhythm (e.g., phase, amplitude, or MESOR), which can impact various functional outputs of the SCN, even if the expression itself is still rhythmic (Vetter, 2020). Several E-box regulated genes (Jin et al., 1999) were increased (*Avp*, *Dbp*, *Nr1d1*, *Ciart*) with chemotherapy. This suggests increased activity of E-box binding elements; however, *Bmal1*, *Clock*, and *Npas2* all had decreased expression magnitudes after chemotherapy. This contradictory pattern of expression represents dysfunctional feedback regulation of E-box–associated elements, which is necessary to maintain an approximate 24 h molecular clock. It is possible that chemotherapy exposure causes DNA damage or other cellular stressors that can alter epigenetic regulation of the transcriptional–translational feedback loop of the molecular clock (Liu et al., 2024), contributing to the enhanced negative feedback control of E-box binding elements observed here. Circadian genes that were predicted to be rhythmically expressed (e.g., *Npas2*) were not significantly rhythmic in either group (He et al., 2022). It is possible that higher resolution of sampling intervals, sampling over multiple circadian cycles, or increasing power would demonstrate the expected rhythmicity. Regardless, several key molecular clock genes (*Bmal1*, *Per2*, *Nr1d1*) were indeed rhythmic after vehicle treatment but disrupted following repeated chemotherapy injections.

While the SCN is a neuron-rich region, other cell types were included in the dissected SCN tissue (Wen et al., 2020). Nevertheless, these transcriptional results imply that the observed circadian disruption occurs in SCN neurons. Here, chemotherapy increased expression of *Avp* in the SCN, which encodes arginine vasopressin (AVP), with no influence on transcription of *Vip* or *Vipr2*, which encodes vasoactive intestinal peptide (VIP) and VIP receptor; expression of AVP receptors V1a and V1b was not measured. The SCN is broadly categorized into core and shell regions, with pacemaking activity beginning in VIP neurons of the core, extending to the AVP neurons of the shell, and then traveling outside of the SCN through humoral and neuronal routes to dictate circadian pacing (Ono et al., 2021; Yamaguchi et al., 2023). Chemotherapy-induced increases of *Avp* that are not concomitant with increased *Vip* could represent decoupling of the SCN core and shell, given that perturbations of AVP activity are associated with decreased core-shell coupling strength and disrupted SCN synchrony (Mieda et al., 2015). Furthermore, chemotherapy abolished rhythmic transcription of *Nms* that encodes a neuropeptide (Neuromedin S) and is rhythmically expressed in VIP and AVP neurons associated with their pacemaking capacity (Lee et al., 2015), further raising the possibility of desynchronized activity either between or within the SCN core and shell neurons.

This SCN molecular clock disruption after paclitaxel chemotherapy corroborates a previous report of damped rhythmic transcription of SCN *Per2* in a mouse model of another chemotherapy treatment, 5-fluorouracil (Terazono et al., 2008). In contrast, the present data contradict other reports indicating no statistically significant disruption of SCN rhythmicity. One study found no changes to period of amplitude of rhythmic PER2 expression in SCN slices after paclitaxel is given in vivo (Sullivan et al., 2022); however, this study had a relatively low sample size and paclitaxel indeed shortened the circadian period of PER2 expression when applied to the SCN culture in vitro. Another chemotherapeutic, doxorubicin, also did not alter SCN rhythmicity as measured by electrophysiology; however, there was misalignment between SCN neuronal activity and behavioral rhythms (Wang et al., 2024). Notably, these reports did not include transcriptional analysis of the SCN-specific tissue and measured these protein/neuronal activity changes over the course of several days following chemotherapy treatment. It is possible that chemotherapy-induced disruption in the SCN only occurs very acutely or exclusively at the transcriptional level. Further work is needed to define rhythmicity of both the transcriptional and translational arms of the SCN molecular clock following distinct types of chemotherapy.

Notably, here chemotherapy resulted in a more stable circadian rhythm immediately following a 6 h phase-delay paradigm compared with vehicle-treated mice, characterized by increased circadian periodogram amplitude and acutely faster entrainment of wheel running offsets (Refinetti, 2004). This contrasted with no changes to entrainment to a phase-advance paradigm after chemotherapy. Though nuanced, these divergent entrainment responses to phase-advance and phase-delay after chemotherapy could demonstrate not only chemotherapy-induced disruption but also identify certain aspects of circadian behavior that are particularly vulnerable to environmental challenges after chemotherapy exposure. While further investigation is needed, the behavioral results here support the aforementioned core-shell decoupling hypothesis. Potentially consistent with our findings, disrupted AVP signaling causes SCN core-shell decoupling and results in faster entrainment to both phase-advance and phase-delay due to a weakened SCN principal clock (Yamaguchi et al., 2013; Rohr et al., 2020). Similarly, knockdown of *Bmal1* in AVP neurons causes blunted light-induced phase-delay at CT14 (Mieda et al., 2015), similar to that observed presently in the PRC after chemotherapy at CT16 and CT20. A decoupled, or weakened, SCN would not be able to robustly respond to a single, unsustained light stimulus and could result in blunted phase-shifts in the PRC. In contrast, a weakened SCN could more easily adjust to sustained re-entrainment paradigms, like jet lag (Mieda et al., 2015). Lastly, the present phase-delay was induced through a long day, whereas the phase-advance was induced through a short night. Modifying the length of day during a phase-shift accelerates entrainment compared with modifying the length of night (Ma et al., 2024). It is possible that if phase-advance was accomplished through a long day rather than a short night, then chemotherapy could have similarly increased re-entrainment as observed with phase-delay.

Importantly, outside of this work, other behavioral phenotypes demonstrating faster re-entrainment with SCN decoupling occur in the context of decreased AVP production and action, but we observed increased expression of SCN *Avp* after chemotherapy. It is possible that while the transcript was increased, the AVP protein may still be decreased (Prabahar et al., 2024). Alternatively, the complex chemotherapy-induced alterations to the SCN molecular clock could result in pleiotropic behavioral outcomes, such as the differing behavioral responses to phase-advance or phase-delay. It is important to note that in our study, due to the possible confound of chemotherapy-induced fatigue acutely after treatment, mice recovered for a week before the circadian light challenges occurred. Additionally, housing conditions can impact circadian rhythms (Robbers et al., 2021), and mice in the present wheel running analyses were singly housed. Thus, direct comparisons between SCN gene expression disruption acutely after chemotherapy and chemotherapy-induced changes to circadian behavioral adaptations are asynchronous. Regardless, the effects of paclitaxel chemotherapy on the SCN circadian transcriptome could indicate a weakened clock, contributing to deficit adaptations to environmental challenges.

Chemotherapy-induced fatigue is a well-characterized phenomenon in both preclinical models and clinical populations and has been associated with circadian disruption (Amidi and Wu, 2022; Tapp et al., 2025). Independently, fatigue is also common following circadian challenges, such as jet lag (Roach and Sargent, 2019). Here, however, neither phase-advance nor phase-delay exacerbated chemotherapy-induced fatigue. In fact, the phase-delay paradigm increased daily activity in chemotherapy-treated mice relative to mice exposed to either phase-delay or chemotherapy alone. It is possible that the phase-delay helped align environmental cues with chemotherapy-induced disruption to the molecular clock, as manipulating environmental light cycles can mask chemotherapy-induced fatigue (Sullivan et al., 2022). Alternatively, chemotherapy and circadian challenges may be contradictory drivers of fatigue, differentially affecting elements of fatigue behavior such as motivation, muscle exhaustion, or sleep disturbances (Harrington, 2012). Further work is needed to better understand this nuanced relationship of fatigue in the face of both treatment- and circadian-associated challenges.

A major remaining question based on the present findings is exactly how chemotherapy is causing disruption at the level of the SCN. Paclitaxel chemotherapy does not readily cross the blood–brain barrier (Angeli et al., 2020) nor would the cytotoxic mechanism of paclitaxel target SCN neurons (Muldoon et al., 2007). However, more work is needed to assess the potential for various chemotherapeutics to enter the SCN parenchyma, such as 5-fluorouracil (Han et al., 2008), and directly elicit circadian disruption. Alternatively, chemotherapy could cause neurotoxicity in inputs to the SCN, such as the ocular nerve or the retinohypothalamic tract, which could impact light information that coordinates circadian rhythms (de Vries et al., 1994; Sodhi et al., 2022). Furthermore, indirect effects of chemotherapy could mediate SCN circadian disruption, such as inflammation. Indeed, paclitaxel increases proinflammatory cytokine production in the SCN (Sullivan et al., 2022), and the molecular clock is sensitive to proinflammatory signaling (Shen et al., 2021). Finally, the presence of tumor alone can impact circadian rhythmicity (Sullivan et al., 2019); thus, additional work is needed to determine how tumor plus chemotherapy may synergize to result in more robust disruption to the SCN and circadian rhythms.

Altogether, these data demonstrate that the SCN molecular clock and its functional output are altered by paclitaxel chemotherapy and contributes to chemotherapy-induced circadian rhythm disruptions. Circadian rhythm disruption is a broad term that can pertain to many different physiological systems (Vetter, 2020) and is linked to several different chemotherapy side effects, such as fatigue and cognitive deficits (Sletten et al., 2020). Understanding specific aspects of circadian physiology that may contribute to circadian rhythm disruption after chemotherapy could pinpoint mechanisms of interest for either pharmacological or noninvasive circadian therapies (e.g., time-restricted feeding and light therapy) to improve patient quality of life during and after treatment.

## Synthesis

Reviewing Editor: Eran Perlson, Tel Aviv University Decisions are customarily a result of the Reviewing Editor and the peer reviewers coming together and discussing their recommendations until a consensus is reached. When revisions are invited, a fact-based synthesis statement explaining their decision and outlining what is needed to prepare a revision will be listed below. The following reviewer(s) agreed to reveal their identity: NONE. Note: If this manuscript was transferred from JNeurosci and a decision was made to accept the manuscript without peer review, a brief statement to this effect will instead be what is listed below.

The authors address all raised concerns.
