# Supplementary figures and images for: Paclitaxel Chemotherapy Disrupts Circadian Gene Transcription and Function of the Suprachiasmatic Nuclei in Female Mice
Source: eNeuro. 2025 Sep 18;12(9):ENEURO.0061-25.2025. doi: 10.1523/ENEURO.0061-25.2025 (PMC12453581; doi:10.1523/ENEURO.0061-25.2025)

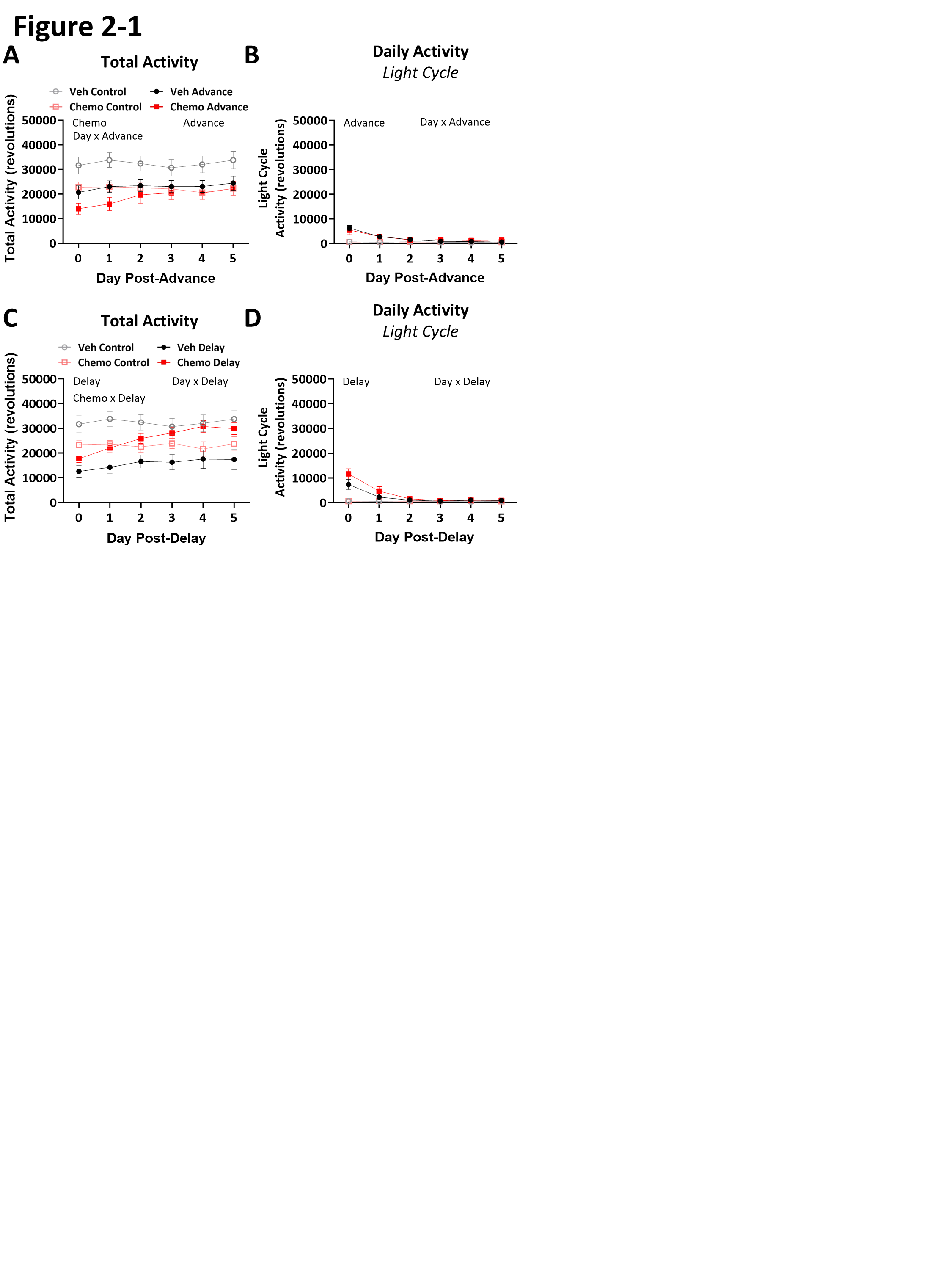

Supplement: Figure 2-1 — Phase-advance and -delay differentially influence total wheel running activity after chemotherapy. (A) Number of daily wheel revolutions acting as an indicator of total activity following phase-advance shift, or relative control period. (B) Number of wheel revolutions that occurred during the light cycle after phase-advance, indicating daily activity during the relative inactive phase. (C) Number of daily wheel revolutions acting as an indicator of total activity following the phase-delay shift, or relative control period. (D) Number of wheel revolutions that occurred during the light cycle after phase-delay, indicating daily activity during the relative inactive phase. N = 9-10/group. Two-way ANOVA main or interaction effects listed on graphs, p < 0.05. Download Figure 2-1, TIF file. [file eneuro-12-ENEURO.0061-25.2025-s003.tif]
