# Supplementary material for: Paclitaxel Chemotherapy Disrupts Circadian Gene Transcription and Function of the Suprachiasmatic Nuclei in Female Mice
Source: eNeuro. 2025 Sep 18;12(9):ENEURO.0061-25.2025. doi: 10.1523/ENEURO.0061-25.2025 (PMC12453581; doi:10.1523/ENEURO.0061-25.2025)
Supplement: Table 1-1 — Chemotherapy alters overall expression of circadian-associated genes regardless of rhythmicity. Download Table 1-1, DOC file. [file eneuro-12-ENEURO.0061-25.2025-s002.doc]

| Table 1-1. Chemotherapy alters overall expression of circadian-associated genes regardless of rhythmicity. | | | |  |
| --- | --- | --- | --- | --- |
| Gene | Rhythmic? | Chemo Effect | *p*-value |  |
|  |
| *Avp* | Chemo | Increase | < 0.0001 |  |
| *Bmal1* | Veh | Decrease | 0.0017 |  |
| *Clock* | N/R | Decrease | 0.0007 |  |
| *Csnk2a1* | N/R | Decrease | 0.0104 |  |
| *Epha6* | N/R | Decrease | 0.0027 |  |
| *Myt1* | Chemo | Decrease | 0.0168 |  |
| *Nfil3* | Chemo | Decrease | 0.0126 |  |
| *Npas2* | N/R | Decrease | 0.0047 |  |
| *Rftn1* | Veh | Decrease | 0.0412 |  |
| *Slc5a3* | Veh | Decrease | < 0.0001 |  |
| *Sytl4* | Chemo | Decrease | 0.0102 |  |
| *Trp53i11* | N/R | Decrease | 0.045 |  |
| Main effects of chemotherapy treatment of SCN genes that are either only rhythmic in one group or not rhythmic in either group. N/R = not rhythmic. | | | |  |
